# Serafino Zappacosta: An Enlightened Mentor and Educator

**DOI:** 10.3389/fimmu.2020.00217

**Published:** 2020-02-13

**Authors:** Ennio Carbone, Mario De Felice, Francesca Di Rosa, Ugo D'Oro, Silvia Fontana, Antonio La Cava, Michele Maio, Giuseppe Matarese, Luigi Racioppi, Giuseppina Ruggiero, Giuseppe Terrazzano

**Affiliations:** ^1^Department of Experimental and Clinical Medicine, University “Magna Graecia” of Catanzaro, Catanzaro, Italy; ^2^Department of Microbiology, Cell and Tumor Biology, Karolinska Intitutet, Stockholm, Sweden; ^3^Istituto per l'Endocrinologia e l'Oncologia Sperimentale, Consiglio Nazionale delle Ricerche (IEOS-CNR), Naples, Italy; ^4^Institute of Molecular Biology and Pathology, Consiglio Nazionale delle Ricerche (IBPM-CNR), Rome, Italy; ^5^GlaxoSmithKline, Siena, Italy; ^6^Department of Medicine, University of California, Los Angeles, Los Angeles, CA, United States; ^7^Center for Immuno-Oncology, Medical Oncology and Immunotherapy, Department of Oncology, University Hospital of Siena, Siena, Italy; ^8^Dipartimento di Medicina Molecolare e Biotecnologie Mediche, Università degli Studi di Napoli “Federico II”, Naples, Italy; ^9^Division of Hematological Malignancies and Cellular Therapy, Department of Medicine, Duke University School of Medicine, Durham, NC, United States; ^10^Dipartimento di Scienze Mediche Traslazionali, Università di Napoli “Federico II”, Naples, Italy; ^11^Dipartimento di Scienze, Università della Basilicata, Potenza, Italy

**Keywords:** education, MHC, T cells, NK cells, immune response

## Abstract

With this article, the authors aim to honor the memory of Serafino Zappacosta, who had been their mentor during the early years of their career in science. The authors discuss how the combination of Serafino Zappacosta's extraordinary commitment to teaching and passion for science created a fostering educational environment that led to the creation of the “*Ruggero Ceppellini Advanced School of Immunology*.” The review also illustrates how the research on the MHC and the inspirational scientific context in the Zappacosta's laboratory influenced the authors' early scientific interests, and subsequent professional work as immunologists.

Serafino Zappacosta, the founder of the “*Ruggero Ceppellini Advanced School of Immunology*” (Ceppellini School), epitomized the term “mentor.” This term was first used by François Fénelon in the book “Les Aventures de Télémaques” to define an enlightened educator who is endowed with unprejudiced knowledge and wisdom (1). The name came after Mentor, the guardian and educator of Odysseus' son Telemachus who offered him encouragement and support for dealing with personal dilemmas while his father was away fighting in the Trojan War. As a mentor and professor of immunology at the University of Naples “Federico II,” Serafino Zappacosta communicated science to his students and close collaborators as a fascinating tool to constantly pursue and advance knowledge, thus nourishing their innate human eagerness to learn. The authors of this article had the privilege to be former members of Zappacosta's laboratory, and wish to offer him this posthumous tribute.

Serafino Zappacosta was a highly cultivated scientist, whose interests spanned from the classics to arts (2). His open-minded vision led him to go beyond the traditional approach to didactics toward new models of education. He conceptualized the idea of an international advanced school of immunology because of his recognition of the quintessential importance in committing educational efforts to nurturing generations of young researchers from all over the world, promoting exchanges between western world and developing countries. The Zappacosta's school model embraced an open and transparent communication that was instrumental to expand the creative potential of independent-minded young investigators, to foster their critical and analytical interests, and to channel their energies into highly valuable scientific directions.

Serafino Zappacosta founded the Ceppellini School in 1991 together with Antonio Di Giacomo (Experimental Immunologist working at the Monaldi Hospital in Naples, Italy), Melchiorre Brai (Professor of Immunology at the University of Palermo, Italy), Giovan Battista Ferrara (Professor of Human Genetics at the Federico II University of Naples, Italy), Albert Nisonoff (Professor of Biology at Brandeis University, in Waltham, Massachusetts, USA), and Ciro Manzo (Head of Immunology Department at the Istituto Pascale in Naples, Italy). The choice of Naples as Ceppellini School headquarters was no accident. This city had experienced in 1799, during the Neapolitan Republic, an unsuccessful attempt to gain freedom from the constraints of a tyrannical monarchy, and to promote a new political organization. This attempt was brutally suppressed. The choice of Naples symbolically reflected the will to propel the freedom of scientific minds according to the School's motto “*non multa sed multum*” (“not many but much,” i.e., quality rather than quantity) (3). Over the years, the Ceppellini School has been attracting and engaging large numbers of international young scientists to the field of immunology.

## Focusing on MHC Molecules

Serafino Zappacosta had a strong interest in the major histocompatibility complex (MHC) genes and proteins. He shared his fascination for the MHC with his laboratory members, with whom he investigated a variety of topics in the MHC field, including the regulation of HLA expression in tumor cells (4, 5), the association between HLA alleles and diseases in Southern Italy (6, 7), the influences of MHC class I (MHC-I) on tumor killing by NK cells (8), and the cytokine-mediated regulation of MHC-I expression (9). His laboratory also worked in collaboration with many international teams to study HLA polymorphisms, and participated in collaborative workshops on HLA typing, that included the International Histocompatibility Workshop in 1991 in Yokohama, Japan (10). This interest in MHC also led to the name of the Ceppellini School, after the immuno-geneticist Ruggero Ceppellini, who had greatly contributed with his pioneer work to the understanding of the genetic bases of HLA polymorphisms, and coined the term “haplotype” (11).

## MHC Polymorphism and the Mediterranean Area

Before molecular genetics could rely on modern technology to collect a tremendous amount of information, many data about the genetic background of different human populations were based on the analysis of products of polymorphic loci including HLA. Analyzing the HLA system at the end of the ‘70s, Zappacosta, together with Mario De Felice, Michele Fiore and Giovan Battista Ferrara (12), found significant differences between people living in Northern Italy and the population of Campania (in Southern Italy). Significant similarities were noticed between Mediterranean and Middle Eastern populations and people from Campania, confirming that the genetic background of the Italian population is highly mixed. Furthermore, a peculiar association between HLA alleles and congenital adrenal hyperplasia was found by Serafino Zappacosta, Michele Maio, Mario De Felice and Rossella Valentino in the Sothern Italian population (6). These studies were performed at the time when Luigi Cavalli-Sforza investigated the selection of advantageous alleles in HLA locus and other polymorphic loci, whereby those findings served to illustrate migration patterns of human populations (13).

Further studies on MHC polymorphism were performed over the years by Giuseppina Ruggiero, Giuseppe Matarese, Giuseppe Terrazzano, and others in the Zappacosta's laboratory. They demonstrated a link between HLA alleles and susceptibility to autoimmune/infectious diseases in Southern Italy (7, 14). Other Zappacosta's team members, including Michele Maio, Luigi Del Vecchio, and Mario De Felice, documented the association between HLA-DR alleles and thyroid carcinoma (15). In the 80', Michele Maio, Luigi Del Vecchio, Giuseppina Ruggiero, Mario De Felice and others of the Zappacosta's laboratory investigated MHC-I expression as a prognostic factor in breast cancer (5). Antonio Pinto, Michele Maio and others showed that HLA-DR expression by myeloid leukemia cells was modulated by anti-neoplastic drugs (4).

## MHC Molecules and the Regulation of NK Cell Response

In the late '80, Silvia Fontana, Luigi Racioppi and Ennio Carbone in Zappacosta's team investigated the link between retroviral infection and MHC-I expression by tumor cells, using virus-induced rat thyroid adenocarcinomas as an experimental model (16, 17). At the time, state-of-the-art techniques for these studies included tissue culture, microscopy, immunofluorescence, and cytofluorimetry, that had only become available a few years earlier. Silvia Fontana, Ennio Carbone, and others in the team showed that tumor transformation modulated MHC-I expression by rat tumor cells, and that rat Large Granular Lymphocytes (LGL) killed more effectively tumor cells having low MHC-I expression (8). These observations were puzzling at that time. In 1987—just a few years before these studies—, Bjorkman and colleagues had solved the HLA A2 crystallographic structure (18, 19), and a lot of attention was concentrated on the TCR/MHC-I molecular interaction, and its role in immunity. On the other hand, Ennio Carbone of the Zappacosta's team was fascinated by pioneering studies on the inhibitory signals provided by MHC-I to Natural Killer (NK) cells [the “missing self” hypothesis, formulated by Klas Karre in 1981 (20)]. Together with Antonio La Cava, Giuseppe Terrazzano and others, the Zappacosta group's contributions to the NK field spanned from MHC-I mediated regulation of NK cell cytotoxicity in rat tumor models (8, 21), to NK cell inhibition induced by soluble HLA-I (22), to new findings on the role of NK cells in human tumor immunosurveillance (23), NK/ dendritic cells cross-talk (24, 25), and CD1-mediated inhibition of NK cytotoxicity (26). Giuseppe Terrazzano in the group investigated the effects of IL-10 on MHC-I expression and on the antigen presenting machinery (9), demonstrating a pathological role of gliadin in the NK cell/ dendritic cells cross-talk (27). Sadly, this publication was the last one that included Serafino Zappacosta's co-authorship.

## MHC Molecules and the Regulation of T Cell Response

In the early 90's, it was well-established that the function of MHC molecules was to bind and present antigenic peptides to T cells (28, 29). The molecular bases of this phenomenon had been largely resolved by several independent studies (30–35). However, looking out of this canonical box, it was possible to hypothesize that, in addition to binding TCR and CD4, MHC class II (MHC-II) molecules might also interact with other cell surface molecules, and in turn regulate the activation of immune cells. Within this context, Zappacosta's group aimed to identify non-canonical functions of HLA-II molecules (36–39). A large panel of monoclonal antibodies (mAbs) directed against different epitopes of HLA-II molecules provided by Soldano Ferrone (40) were instrumental for these studies, that were performed in *in vitro* models of polyclonal T-cell proliferation, induced by either phytohemoagglutinin (PHA) or anti-CD3 mAb (41, 42). Although the presence of antigen presenting cells (APC) was required to achieve full T-cell activation, HLA-II antigen presenting function was largely dispensable in these models, thus offering a unique opportunity to evaluate non-canonical functions of HLA-II molecules.

Giuseppina Ruggiero and Luigi Racioppi in Zappacosta's team, in collaboration with Ciro Manzo, initiated these pioneer studies on HLA-II molecules, and demonstrated that the incubation of autologous monocyte-derived macrophages with mAbs directed against non-polymorphic determinants of HLA-II molecules exerted a remarkable inhibitory effect on T cell activation (37, 38, 43). This result suggested that MHC-II molecules expressed on the APC could interact not only with the TCR and CD4, but also with additional ligand(s)—at the time unknown—, expressed on the T cell surface. Interestingly, in 1996 one of these hypothetical ligand was identified by Huard et al. (44), who demonstrated the ability of CD223 (aka LAG-3) to bind MHC-II molecules. In the last two decades, the relevance of LAG-3/MHC-II signaling has been confirmed by several independent studies, being this molecular interaction involved in a variety of immuno-regulatory circuits (45).

D'Oro and Di Rosa from the Zappacosta's team further explored non-canonical functions of MHC-II molecules expressed by activated human T cells. These studies were based on the general hypothesis that HLA-II molecules might transduce intracellular signals, and in turn finely tune T cell response to antigen(s) and cytokines. The results confirmed this possibility, showing that cross-linking of HLA-II on activated T cells was sufficient to induce inositol triphosphates (IP3) accumulation, protein kinase C (PKC) activation, and, ultimately, enhanced T cell proliferation (10, 39). Of note, the ability of MHC-II molecules to transduce intracellular signals has also been recognized in B cells (46–48), and more recently a cell-intrinsic contribution of MHC-II expression has been shown in the B cell development in the bone marrow (49).

As a note, the findings by Zappacosta's group on non-canonical functions of the HLA molecules have relevant, and still largely unexplored, implications in the regulation of the human immune response. For example, high expression of LAG-3 by T regulatory (Treg) cells suggests that LAG-3/MHC-II complexes might play an important role in the bi-directional signaling triggered by Treg/ T effector cell interactions (50, 51). In this sense, an increasing number of studies has pointed to LAG-3/MHC-II interaction as an attractive druggable target to treat autoimmune diseases, stimulate anti-cancer immune response (52), and revert resistance to anti-PD-1 immunotherapy (53).

## MHC and Beyond

Giuseppe Matarese devoted his experimental efforts on the innovative hypothesis that nutrient-energy-sensing pathways might represent a powerful tool to control immunological self-tolerance. He showed that leptin, a hormone critically involved in energy balance and body weight regulation, acts as a strong immune-modulator, that influences the susceptibility to infection and autoimmunity (54, 55). Leptin levels inversely correlated with regulatory T cell number in multiple sclerosis patients (56), and a direct link between leptin and regulatory T cell anergy was established (57). This observation, that was further developed in subsequent studies performed by Giuseppe Matarese and his group, in collaboration with Antonio La Cava, was the result of frequent, endless, unforgettable evening lab discussions with Serafino Zappacosta.

After training with Serafino Zappacosta with a focus on the immune-modulating properties of MHC molecules, the team members subsequently developed new hypotheses and investigations in diverse directions, ranging from the study of the fundamental mechanisms of immune regulation and immunological memory, to autoimmunity, cancer immunotherapy and vaccinology (58–66). Serafino Zappacosta kept to enthusiastically follow the progress of the past members of his team after they left to start their independent careers. Many of them remained involved over the years in the Ceppellini School activities, either as faculty members or as components of the board of directors, maintaining the School as an arena of continuous scientific education and dynamic discussion.

## Conclusions

The review summarizes the legacy left by Serafino Zappacosta to his collaborators who, albeit with different individual perspectives and at a different degree, continued to work on the MHC, looking at these molecules as a window of opportunity to comprehend the complexity of the immune response, rather than merely looking at them as antigen presenting molecules.

After the death of Serafino Zappacosta in 2006, Silvia Fontana became the President of the Ceppellini School, and Ennio Carbone, Giuseppe Matarese, Francesca Di Rosa, Giuseppina Ruggiero, Giuseppe Terrazzano and other previous collaborators of the Zappacosta's team continued to organize advanced international immunology courses, together with the long-standing Serafino Zappacosta's collaborators and friends Antonio Di Giacomo, who co-founded the School in 1991 (3), Elizabeth Simpson, who organized the first Ceppellini School Course in 1992 on bone marrow transplantation (67), and the newly recruited Ceppellini School Scientific Director Stefan Kaufmann (68). A new type of event, the Serafino Zappacosta Memorial Conferences, was initiated in 2007. Since 2010, this event has been held in the newly inaugurated “Serafino Zappacosta” Auditorium of the Federico II University of Naples. All these activities were made possible by the excellent work of the Ceppellini School Scientific Secretary Tricia Reynolds.

To conclude, the continuation of the activities of the Ceppellini School not only allows an unceasing engagement of new young bright minds to the fascinating field of immunology, but also keeps alive Serafino Zappacosta's dream that intellectual freedom can be shared without boundaries for the benefit of younger generations.

## Author Contributions

All authors listed have made a substantial, direct and intellectual contribution to the work, and approved it for publication.

## Dedication

This article is dedicated to the memory of Luigi Del Vecchio, past member of Zappacosta's team and full professor of Clinical Biochemistry and Molecular Biology, Dipartimento di Medicina Molecolare e Biotecnologie Mediche, Università degli Studi di Napoli “Federico II,” Naples, Italy at the time of his premature death in 2018.

### Conflict of Interest

UD is an employee of the GSK group of companies and holds restricted shares of the GSK group of companies. The remaining authors declare that the research was conducted in the absence of any commercial or financial relationships that could be construed as a potential conflict of interest. The reviewer DB declared a shared affiliation, with no collaboration, with several of the authors, MD, FDR, SF, to the handling editor at time of review.
